# Efficacy and moderators of isometric resistance training (IRT) on resting blood pressure among patients with pre- to established hypertension: a multilevel meta-review and regression analysis

**DOI:** 10.1186/s13102-025-01286-0

**Published:** 2025-08-16

**Authors:** Chengyu Zhou, Sijia Li, Zengyu Zhang, Jiamin Chen, Jian Song, Yongmin Xie, Zhuolin Tang, Aiguo Zhou

**Affiliations:** 1 Strength and Conditioning Training College, Beijing Sports University, Beijing, China; 2 College of Sports Medicine and Rehabilitation, Beijing Sport University, Beijing, China; 3 Research Center for Clinical Medicine, Jinshan Hospital Affiliated to Fudan University, Shanghai, China; 4 College of Integrative Chinese and Western Medicine, Hunan University of Traditional Chinese Medicine, Changsha, Hunan China; 5 School of Physical Education, Shanghai Normal University, Shanghai, China; 6 School of Physical Education, Sichuan Agricultural University, Yaan, Sichuan China

**Keywords:** IRT, Blood pressure, Meta, Base, Hypertension

## Abstract

**Objective:**

This study aims to evaluate the efficacy of isometric resistance training (IRT) in lowering blood pressure through meta-analyses, specifically systolic blood pressure (SBP), diastolic blood pressure (DBP), and mean arterial pressure (MAP), in individuals with pre-hypertension to established hypertension. Additionally, it seeks to identify potential moderators that influence the blood pressure–lowering effects of IRT.

**Methods:**

A systematic search was conducted in Web of Science (Core Collection), PubMed, Cochrane Library, and CNKI to identify studies employing a between-group design in individuals with pre-hypertension to established hypertension that assessed the effects of IRT on blood pressure. The initial search was performed in February 2024 and updated in May 2025. A multilevel meta-analysis was conducted to pool effect estimates for each outcome, reported as mean difference (MD) and standardized mean difference (SMD). Heterogeneity was examined using subgroup analyses, meta-regression, and sensitivity analyses.

**Results:**

The present meta-review included 30 original studies, and meta- analysis results suggested compared to the control group, IRT significant reductions in SBP by 7.31 mmHg (SMD = -0.76, I² = 75%, moderate certainty of evidence), DBP by 3.90 mmHg (SMD = -0.54, I² = 48%, low certainty of evidence), and MAP by 5.94 mmHg (SMD = -0.92, I² = 53%, moderate certainty of evidence) among individuals with pre-hypertension to established hypertension (*p* < 0.01 for all). Sensitivity analysis confirmed that the above combined results were stable and there was no risk of publication bias. Subgroup analyses identified region, health status, medication use, training mode, interval duration, and frequency as significant moderators of IRT’s effect on SBP, while BMI, interval duration, and frequency significantly moderated its effect on DBP (*p* < 0.05 for all). Meta-regression showed that SBP reduction was associated with age, female proportion, and training intensity (%MVC), with each 10% MVC increase in intensity linked to a ~ 2 mmHg SBP and ~ 1.3 mmHg MAP reduction in the range 10–60% MVC. For DBP, age was the only significant predictor.

**Conclusion:**

IRT was efficacious in reducing resting SBP, DBP, and MAP in individuals with pre-hypertension to established hypertension. The antihypertensive effects of IRT may be moderated by factors such as age, sex, medication status, baseline BP, and training variables (e.g., intensity, modality, recovery), underscoring the need for individualized and well-controlled applications in practice and research. A protocol recommends IRT at 30–60% MVC (gradually increase) or wall squat IRT at approximately 85%-95% HRpeak, performed 3–5 sessions per week for 14 min per session (4 × 2 min contractions with 2 min recovery).

**Supplementary Information:**

The online version contains supplementary material available at 10.1186/s13102-025-01286-0.

## Introduction

Cardiovascular diseases persist as a leading cause of global mortality and morbidity. Hypertension emerges prominently as the primary modifiable risk factor within cardiovascular health, closely linked to heightened occurrences of myocardial infarction, stroke, and heart failure [1]. Epidemiological surveys underscore a continual upward trajectory in hypertension prevalence in recent years, affecting a minimum of 523 million individuals and contributing to 18.6 million deaths [1]. This pervasive prevalence places a substantial economic burden on societies [2]. Key contributors to the onset of hypertension include insufficient physical activity, sedentary behavior [3], excessive dietary salt intake, overweight or obesity, smoking, and alcohol consumption [4]. Nevertheless, contemporary research reveals that even with a blend of pharmaceutical interventions and adjunctive therapies, attaining optimal blood pressure control remains a formidable challenge [5]. Consequently, national and international hypertension prevention and treatment guidelines underscore non-pharmacological lifestyle modifications as primary interventions [6].

As a pivotal non-pharmacological lifestyle intervention, exercise has progressively attracted attention due to its potential to mitigate blood pressure in pre- to established hypertension [7]. Traditionally, the most widely recognized form of exercise is moderate-intensity continuous training (MICT), credited with reducing resting blood pressure by 8.9 mmHg in hypertensive subjects [8]. This approach is further enriched by the inclusion of resistance training within the exercise regimen, as advocated [9], and it has been widely shown to be beneficial in the management of cardiovascular diseases [10]. However, recent research has systematically evidenced that diverse forms of exercise may manifest varying impacts on the reduction of blood pressure and suggested novel evidence. A comprehensive network meta-analysis, encompassing 270 randomized controlled trials (RCTs) and involving 15,827 participants, has unveiled that isometric resistance exercise emerges as particularly efficacious in the reduction of resting blood pressure [11]. Additionally, this subgroup analysis provides empirical support for the effectiveness of integrating daily, highly practical isometric squatting as a viable application in real-world scenarios. Moreover, direct “head-to-head” comparisons between isometric resistance training and high-intensity interval training, meta-analyses have consistently affirmed the superior efficacy of IRT in blood pressure management [12]. Given the current low utilization rate of gyms [13], a single IRT session lasting 11 to 20 min, its applicability in various environments, and lower equipment costs make it a potentially advantageous type of training [14].

Hence, IRT emerges as a potentially more effective exercise intervention for individuals with pre- to established hypertension. The current meta-analysis of the efficacy of IRT in hypertensive patients still has inconsistencies and limitations. Notably, Almeida et al. conducted a meta-analysis that included 5 studies that showed IRT had no impact on diastolic blood pressure (DBP) in hypertensive subjects (mean difference [MD] = -2.75 mmHg, *p* = 0.42) [15]. In contrast, Baffour-Awuah et al. (2023) included 12 studies that suggested the efficacy of IRT in reducing DBP (MD = -3.17 mmHg, *p* < 0.01) [16]. Moreover, these meta-analyses lacked a comprehensive evaluation of studies involving individuals with prehypertension or at elevated cardiovascular risk [15], and may have excluded Chinese-language studies due to language restrictions. This omission potentially compromises the global representativeness and comprehensiveness of the evidence base. Additionally, both reviews failed to include several earlier original studies [17–21], and with the emergence of numerous new trials in recent years [22–26], an updated meta-analysis encompassing both prehypertensive and hypertensive populations is warranted. Such an effort can offer timely, evidence-based insights to reconcile existing inconsistencies and controversies. Furthermore, we will reassess the quality of the evidence, addressing concerns that earlier meta-analyses may have overestimated it by neglecting the evaluation of risk of bias and failing to account for heterogeneity in pooled estimates [16].

Notably, previous meta-analyses have not thoroughly explored moderators influencing the antihypertensive effects of IRT in hypertensive individuals [15, 16, 27]. This gap limits the precision of exercise prescription guidelines. In response, our review will provide evidence-based recommendations on key training parameters—such as intensity, duration, rest intervals, modality, and frequency—through subgroup and meta-regression analyses. While Baffour-Awuah et al. (2023) made efforts to investigate the moderating of specific factors through subgroup analyses, certain methodological limitations persist, such as utilizing intervention sessions in subgroup analysis instead of the more precise and recommended meta-regression analysis [16]. In light of these unresolved issues and limitations, an updated and methodologically rigorous meta-analysis is essential. Employing advanced statistical approaches, such as three-level modeling and robust variance estimation, combined meta-regression to explored potential dose- response relations will help refine and enhance the quality and applicability of evidence for the use of IRT in blood pressure management.

The present study aimed (1) to conduct a meta-analysis review quantifying the effects of IRT on SBP, DBP, and mean arterial pressure (MAP) in adults with pre- to established hypertension and (2) to identify and examine potential moderating factors influencing these effects.

## Methods

This review was performed following the Preferred Reporting Items for Systematic Reviews and Meta-Analyses (PRISMA) guidelines [28], and the completed PRISMA 2020 checklist can be found in Appendix 1. This review was registered in the PROSPERO database, given the identifier CRD4202451625.

### Search strategy

The two researchers (JMC and ZYZ) conducted a systematic search in databases including the Web of Science (Core Collection), PubMed, Cochrane Library, and CNKI with a search period from February 2024, and a second search update was conducted in May 2025 to ensure the inclusion of the most recent publications. The search terms comprised keywords and medical subject headings. Using the Web of Science as an example, the search strategy was as follows: ((“IRT” OR “isometric resistance training” OR “isometric training” OR “isometric exercise”) AND (“blood pressure” OR “systolic pressure” OR “diastolic pressure” OR “MAP” OR “arterial pressure” OR “mean arterial pressure” OR “hypertens*” OR “antihypertens*” OR “arterial pressure” OR “hypertension” OR “prehypertension”)). Additionally, this study reviewed the reference lists of relevant meta-analyses and literature.

### Study selection

This study utilized EndNote X9 [Clarivate Analytics, 2018] to deduplicate the literature. Subsequently, two researchers (JMC and ZYZ) independently reviewed the titles and abstracts of the literature according to predefined inclusion and exclusion criteria. In case of discrepancies during the review process, the researchers would convene a meeting to discuss the issues about the established criteria to reach a consensus. If a consensus still could not be reached, a third researcher (CYZ) would be invited to participate, ultimately deciding whether the literature met the inclusion criteria. In the full text review phase, the two researchers (JMC and ZYZ) would also proceed independently, and the same method used in the title and abstract screening stage would be applied to address any discrepancies that arise.

### Eligibility criteria

We followed the PICOS (participants, interventions, comparators, outcomes, and study design) approach [28] and studies eligible for inclusion had to meet the following criteria: (1) population: As the population participants aged 18 years or older with pre-hypertension (office SBP values ≥ 130 mmHg and/or DBP values ≥ 85mmHg) or hypertension (office SBP values ≥ 140 mmHg and/or DBP values ≥ 90 mmHg) based on the classification of the European Society of Cardiology and the European Society of Hypertension guidelines. (2) Intervention: we considered studies of IRT at above 10% MVC delivered for a minimum of 2 weeks or six sessions. (3) Comparator: the comparator involved non-training control groups. (4) Outcome: outcome measures were blood pressure, including systolic, diastolic, and mean arterial pressure. (5) Study design: We included literature-type controlled trials, whether parallel or vertically crossed, and whether randomized or not.

### Risk of bias assessment

The risk of bias in the studies was evaluated independently by two reviewers using the Cochrane Risk of Bias tool. This tool consists of several predefined domains of bias, including selection bias, performance bias, detection bias, attrition bias, reporting bias, and other biases. The assessment of “other” bias involved determining if the studies incorporated supervised or monitored exercise interventions and reported exercise adherence. Each domain was rated as low, unclear, or high risk of bias. In cases of disagreement, a third reviewer resolved the discrepancies.

### Data extraction

In this study, all eligible research data were independently extracted by two reviewers (SJL and ZLT) and recorded in a predetermined form. A third reviewer (CYZ) was responsible for cross-verifying the extracted data. The extracted information included the lead author’s name, publication year, participant characteristics, study design, training protocol, type of exercise testing used to assess blood pressure (BP), training mode, and pre- and post-intervention means and standard deviations of BP.

This study extracted the mean, standard deviation, and sample size reported for each group pre- and post-intervention. We pooled effects using pre- and post-intervention differences (M ± SD) for each outcome indicator. The first step was to calculate the difference in means (raw mean difference between post and preintervention for each intervention group):

1 $$\:{MD}_{diff}\:=\:{M}_{post}\:-\:{M}_{pre}$$

where $\:{MD}_{diff}\:$the raw mean difference, $\:{M}_{post}$ is the reported mean post-intervention, and $\:{M}_{pre}$ is the reported mean pre-intervention.

If the study only reported confidence intervals, they were converted to *SD* using the following formula:

2 $$\:SD=\sqrt{N}\:\frac{{CI}_{ℎigℎ}-\:{CI}_{low}}{2t}$$

where *SD* is the standard deviation, *N* is the group sample size, $\:{CI}_{ℎigℎ}\:$is the upper limit of the confidence interval, $\:{CI}_{low}$ is the lower limit of the confidence interval, and *t* is the *t* distribution with *N* − 1 degrees of freedom the respective confidence level.

The *SD* of the difference in means ($\:{SD}_{diff}$) was calculated as follows [29]:

3 $$\:{SD}_{diff}=\sqrt{{{SD}_{pre}}^{2}+{{SD}_{post}}^{2}-2r\times\:{SD}_{pre}\times\:{SD}_{post}}$$

where $\:{SD}_{diff}\:$is the standard deviation of the difference in means, $\:{SD}_{pre}$ is the standard deviation from pre-intervention, and $\:{SD}_{post}\:$is the standard deviation from post-intervention. As the original studies included in the meta-analysis did not report Pearson’s correlation coefficients (r) for pre- and post-intervention outcomes, we first attempted to use *r* = 0.53 by O’Driscoll et al., (2022) studies [30] that reported a $\:{SD}_{cℎange}$ based on the recommendations in the Cochrane.

4 $$\:r=\frac{{{SD}_{pre}}^{2}+{{SD}_{post}}^{2}-{{SD}_{cℎange}}^{2}}{2\times\:{SD}_{pre}\times\:{SD}_{post}}$$

The formula for merging subgroups is as follows: Assume that the sample size of subgroup A is *N*_*1*_, the mean is *M*_*1*_, and the standard deviation is *SD*_*1*_; the sample size of subgroup B is *N*_*2*_, the mean is *M*_*2*_, and the standard deviation is *SD*_*2*_, then the combined Sample size *N = N*_*1*_ *+ N*_*2*_, mean *M=(N*_*1*_*M*_*1*_ *+ N*_*2*_*M*_*2*_*)/(N*_*1*_ *+ N*_*2*_*)*. The formula for pooled standard deviation is as follows [29]:

5 $$\:SD=\sqrt{\frac{\left({N}_{1}-1\right){S}_{1}^{2}+\left({N}_{2}-1\right){S}_{2}^{2}+\frac{{N}_{1}{N}_{2}}{{N}_{1}+{N}_{2}}({M}_{1}^{2}+{M}_{2}^{2}-2{M}_{1}{M}_{2})}{{N}_{1}+{N}_{2}-1}}$$

### Data synthesis and statistical analysis

Statistical analyses were conducted using the “metafor” package in R (version 4.2.1) [31]. Given that some included studies reported multiple experimental groups, which could lead to the repeated inclusion of a single study in the combined model and artificially inflate the overall effect size in a traditional two-level meta-analysis [32], we employed a three-level meta-analytic approach as described by Assink and Wibbelink [33]. This model partitions variance into three components: sampling variance (Level 1), within-study variance (Level 2), and between-study variance (Level 3), thereby accounting for the nested structure and correlation among effect sizes [34].

To further address potential dependencies among effect sizes within studies, a variance-covariance matrix-based clustering-robust variance estimation (CRVE) was applied, with small-sample corrections to ensure unbiased estimation under correlated data conditions [35]. By retaining multiple effect sizes from the same study, the three-level model increases statistical power and provides more precise estimates [33]. In addition, to account for expected heterogeneity arising from variations in study design, interventions, and populations, we adopted a random-effects model [36], which assumes that true effects are normally distributed across studies. Parameter estimates were obtained using restricted maximum likelihood (REML), and results were cross-validated using the maximum likelihood (ML) method to ensure robustness.

Considering the clinical relevance and magnitude quantification, we will calculate both mean difference and standardized mean difference (SMD) [37] as the effect size indicator. Additionally, considering the small sample sizes in most included studies, this study used Hedges’ g, based on the precise formula, as the effect size, and corrections for bias were applied. The Hedges’ g values were categorized as *trivial* (0.2), *small* (0.2–0.5), *medium* (0.5–0.8), or *large* (> 0.8).

A variety of metrics, including Cochrane’s Q, I^2^ statistic, tau^2, and Tau, were employed to evaluate heterogeneity [38]. We computed a 95% confidence interval (CI). The prediction interval (PI) was also calculated to more comprehensively reflect the potential variability in future similar studies, and multiple indicators were reported simultaneously [37]. Recent literature and standard textbooks predominantly advocated for the I^2^ statistic as the principal indicator of heterogeneity. Consequently, our primary analysis utilized I^2^, interpreting its values as follows: 0%-25% potentially insignificant; 25%-50% indicative of moderate heterogeneity; 50%-75% suggestive of substantial heterogeneity; 75%-100% reflective of considerable heterogeneity [39].

Additionally, the statistical power of the primary pooled effect was calculated, and the possibility of false negatives due to insufficient statistical power was considered. Statistical power calculations were performed using the *metameta* package [40].

To investigate the sources of heterogeneity between studies and moderating factors, this study employed subgroup and meta-regression analysis to conduct statistical analyses on binary and continuous variables [41]. Subgroup analyses were conducted based on a comprehensive set of variables, which were categorized into three main domains to better interpret the potential moderators of IRT efficacy. Participant characteristics included region, BMI category, hypertension condition, and medication status. IRT training characteristics encompassed whether the intervention was supervised, the mode of training, training laterality, interval duration, and training frequency. Study design characteristics included whether the trial employed randomization. The meta-regression was performed to further explore potential continuous moderators of the blood pressure–lowering effects of IRT. The regression models incorporated variables across both participant and intervention domains. Participant-level variables included age and the proportion of female participants. Training-related variables comprised IRT intensity (expressed as %MVC; studies that quantified intensity using %HR_peak_ were excluded from the regression to ensure consistency), total session duration, total weekly duration, total intervention duration (weeks), and cumulative training time. It is recommended that at least 10 studies be available for each meta-regression (minimum of five studies per group for subgroup analyses [42]. The regression analysis was based on a mixed-effect model, calculated using restricted maximum likelihood estimation, which is generally considered more robust than traditional maximum likelihood estimation as it accounts for the presence of fixed effects when estimating random effects.

Lastly, this study carried out sensitivity analyses and used exclusion methods to identify individual case studies that might have caused differences in the overall results. Additionally, this study used the contour-enhanced funnel plot and Egger’s asymmetry test [43] to check for publication bias. A *p*-value > 0.05 indicated no publication bias.

### Certainty of the evidence

Evidence of effectiveness for each study was combined with quality scores for use in discussing the results. The Grading of Recommendations Assessment, Development, and Evaluation (GRADE) methodology was used to rate the certainty of the evidence as “high”, “moderate”, “low” or “very low” [44]. GRADE was completed by two researchers, with differences resolved through consensus. This comprehensive assessment rates evidence as follows: (1) the risk of bias, downgraded by one level if “some concerns” and two levels if “high risk” of bias; (2) inconsistency, downgraded by one level when the impact of statistical heterogeneity (I2) is moderate (> 25%) and by two levels when high > 75%; (3) imprecision: downgraded by one level when statistical power < 80% and if there was no clear direction of the effects [45]; (4) risk of publication bias: downgrade one level if Egger’s test < 0.05.

## Results

### Studies retrieved

**Fig. 1:**
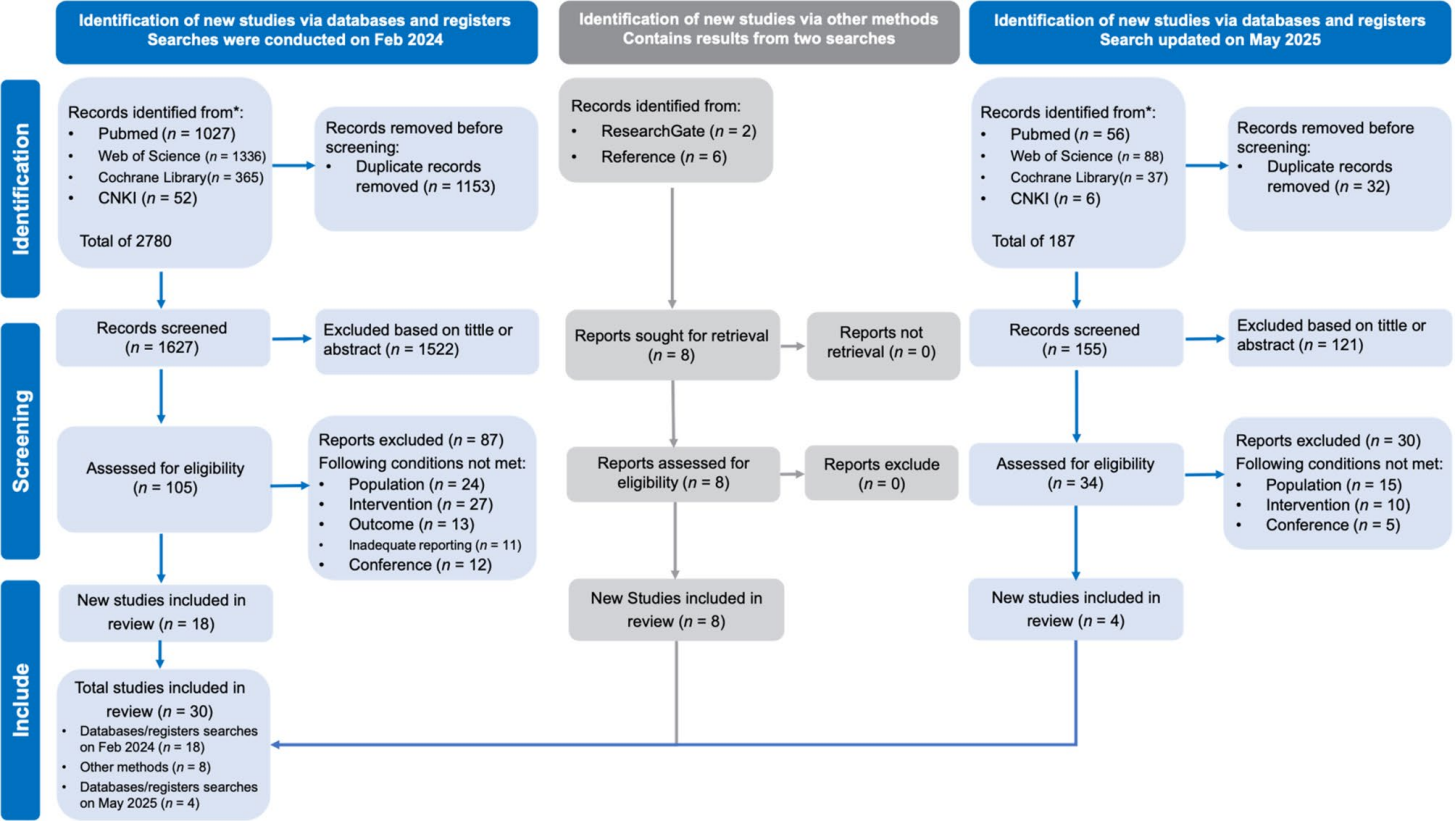
PRISMA flow diagram for included and excluded study

The initial search conducted in February 2024 identified 2,780 records. After screening, 18 studies met the inclusion criteria for the meta-analysis. An updated search was performed in May 2025, which yielded an additional 12 eligible studies. Ultimately, a total of 30 studies were included in the present meta-analysis [17–26, 46–65]. The detailed selection process is illustrated in Fig. 1.

### Characteristics of included studies

Table 1 lists the characteristics of these studies. In total, thirty studies were included. Six studies were from North America, five studies were from South America, eleven studies were from Europe, one study was from Africa, and seven studies were from Asia. The subjects in these studies were approximately 40% female, with an average age of approximately 60 years and an average BMI of approximately 27.6 kg/m^2^. Eight studies did not report BMI, one study did not report gender, and participants in six studies were only male. Eleven studies included participants who did not take medication, and ten studies included participants with pre-hypertension, while the remaining studies were mainly patients with established hypertension taking medication. Table 1 also summarizes the characteristics of the interventions. Fifteen studies used unsupervised intervention; twenty-three studies used hand exercises; seven studies used squatting; and one study simultaneously used hand exercises and squatting. The training intensity of IRT training performed with hands was 10–60% maximum voluntary contraction (MVC), and the training intensity of IRT training performed with legs was 85–95% HRpeak. Most studies used 4 sets × 2 minutes of formal training, with only one study using 4 sets × 0.75 min of formal training. The duration ranged from 3.4 to 48 weeks, with 8-week interventions being the most common. The frequency of exercise ranged from 3 to 7 times per week, with only one study using a training frequency of 1 session per day, and the remaining studies all used a training frequency of 3 times per week.

**Table 1:** Summary of included study characteristics

Study	Region	K	Age	BMI	Female Rate (%)	Health and Medication	Intervention	Outcome
Badrov 2013 [47]	North America	24	65	28	46	1)Established Hypertension; 2) medicated	1) Supervised 2) Hand, 4$\:\times\:$2 min, recovery 1 min, 30% of MVC, 3 session per week, 10 week	SBP, DBP, MAP
Baross 2012	UK	30	53.9	n/a	0	1) Pre-hypertension 2) No-medicaed	1) Supervised 2) Squat, 4$\:\times\:$2 min, recovery 2 min, 11% of MVC, 3 session per week, 8 week	SBP, DBP, MAP
Baross 2013	UK	20	54	n/a	0	1) Pre-hypertension 2) n/a	1) Supervised 2) Squat, 4$\:\times\:$2 min, recovery 2 min, 85% HR, 3 session per week, 8 week	SBP, DBP, MAP
Cahu Rodrigues 2020 [50]	South America	33	53	31	70	1)Established Hypertension; 2) medicated	1) Supervised 2) Hand, 4$\:\times\:$2 min, recovery 1 min, 30% of MVC, 3 session per week, 12 week	SBP, DBP
Danielsen 2023 [22]	European	48	64	26.5	37.5	1)Established Hypertension; 2) medicated	1) Unsupervised 2) Hand, 4$\:\times\:$2 min, recovery 1 min, 30% of MVC, 3 session per week, 20 week	SBP, DBP
Correia 2020 [51]	North America	79	67	26.5	34	1)Established Hypertension; 2) medicated	1) Unsupervised 2) Hand, 4$\:\times\:$2 min, recovery 4 min, 30% of MVC, 3 session per week, 8 week	SBP, DBP
Cohen 2022	Colombia	77	44.9	28.1	33	1)Established Hypertension 2) No-medicated	1) Supervised 2) Hand, squat, 4$\:\times\:$2 min, recovery 2 min, 30% of MVC, 3 session per week, 12 week	SBP, DBP
Edwards 2023	UK	24	43.8	n/a	0	1) Pre-hypertension 2) No-medicated	1) Unsupervised 2) Squat, 4$\:\times\:$2 min, recovery 2 min, 95% HR, 3 session per week, 4 week	SBP, DBP
Farah 2018 [52]	South America	40	59	29.9	69	1)Established Hypertension; 2) medicated	1) Supervised/ Unsupervised 2) Hand, 4$\:\times\:$2 min, recovery 1 min, 30% of MVC, 3 session per week, 12 week	SBP, DBP, MAP
Fecchio 2023 [24]	South America	35	55	29	0	1)Established Hypertension; 2) medicated	1) Supervised 2) Hand, 4$\:\times\:$2 min, recovery 1 min, 30% of MVC, 3 session per week, 10 week	SBP, DBP
Gordon 2018 [53]	North America	22	49	26	73	1)Established Hypertension 2) medicated	1) Supervised/ Unsupervised 2) Hand, 4$\:\times\:$2 min, recovery 1 min, 30% of MVC, 3 session per week, 12 week	SBP, DBP
Herrod 2020	UK	23	71	n/a	52	1) Pre-hypertension 2) medicated	1) Supervised 2) Hand, 4$\:\times\:$2 min, recovery 2 min, 30% of MVC, 3 session per week, 6 week	SBP, DBP
Javidi 2022	Iran	39	46.1	n/a	0	1)Established Hypertension 2) No-medicated	1) Supervised 2) Hand, 4$\:\times\:$2 min, recovery 4 min, 45% of MVC, 3 session per week, 8 week	SBP, DBP, MAP
Lea 2024 [55]	Europe	30	40	28	20	1) Pre-hypertension 2) No-medicaed	1) Unsupervised 2) Squat, 4$\:\times\:$2 min, recovery 2 min, 95% HR_peak_, 3 session per week, 4 week	SBP, DBP, MAP
Millar 2013 [19]	North America	23	65	27	22	1)Established Hypertension 2) medicated	1) Supervised 2) Hand, 4$\:\times\:$2 min, recovery 4 min, 30% of MVC, 3 session per week, 8 week	SBP, DBP, MAP
Nemoto 2025	Japan	56	66.9	24.6	33.1	1) Pre-hypertension 2) medicated	1) Unsupervised 2) Hand, 4$\:\times\:$2 min, recovery 1 min, 15% of MVC, ≥ 3 session per week, 12 week	SBP, DBP
Nemoto 2021 [57]	Asian	53	61.7	25	43.4	1)Established Hypertension 2) medicated	1) Unsupervised 2) Hand, 4$\:\times\:$2 min, recovery 1 min, 30% of MVC, 3 session per week, 8 week	SBP, DBP
O’Driscoll 2022 [20]	Europe	24	45	27.7	0	1)Established Hypertension 2) No-medicaed	1) Unsupervised 2) Squat, 4$\:\times\:$2 min, recovery 2 min, 95% HR_peak_, 3 session per week, 48 week	SBP, DBP
Ogbutor 2019	Nigeria	400	40	29.38	47	1) Pre-hypertension 2) No-medicaed	1) Supervised 2) Hand, 2$\:\times\:$2 min, recovery 5 min, 30% of MVC, 1 session per day, 24 day	SBP, DBP
Okamoto 2022	Japan	22	75	21.6	59	1) Pre-hypertension 2) No-medicaed	1) Unsupervised 2) Hand, 4$\:\times\:$2 min, recovery 1 min, 30% of MVC, 5 session per week, 8 week	SBP, DBP, MAP
Okamoto 2020 [60]	Asian	22	65	n/a	59	1)Established Hypertension 2) No-medicaed	1) Unsupervised 2) Hand, 4$\:\times\:$2 min, recovery 1 min, 30% of MVC, 5 session per week, 8 week	SBP, DBP
Pagonas 2017	Germany	47	60.5	26.8	60	1)Established Hypertension 2) n/a	1) Supervised 2) Hand, 4$\:\times\:$2 min, recovery 1 min, 30% of MVC, 5 session per week, 12 week	SBP, DBP
Palmeira 2021 [61]	South America	31	55	30	70	1)Established Hypertension 2) medicated	1) Supervised 2) Hand, 4$\:\times\:$2 min, recovery 1 min, 30% of MVC, 3 session per week, 12 week	SBP, DBP
Pinto 2025	Portugal	76	71.3	28.6	53	1)Established Hypertension 2) medicated	1) Unsupervised 2) Hand, 4$\:\times\:$0.75 min, recovery 1 min, 50% of MVC, 3 session per week, 8 week	SBP, DBP, MAP
Punia 2020 [62]	Asian	40	37.5	n/a	50	1) Pre-hypertension 2) medicated	1) Unsupervised 2) Hand, 4$\:\times\:$2 min, recovery 4 min, 30% of MVC, 3 session per week, 8 week	SBP, DBP, MAP
Stiller-Moldovan 2012 [63]	North America	20	61.5	31	50	1)Established Hypertension 2) medicated	1) Supervised 2) Hand, 4$\:\times\:$2 min, recovery 1 min, 30% of MVC, 3 session per week, 8 week	SBP, DBP, MAP
Taylor 2003 [64]	North America	17	67.5	n/a	41	1)Established Hypertension 2) medicated	1) Supervised 2) Hand, 4$\:\times\:$2 min, recovery 1 min, 30% of MVC, 3 session per week, 10 week	SBP, DBP, MAP
Taylor 2019 [65]	Europe	48	43.8	28.3	n/a	1) Pre-hypertension 2) No-medicated	1) Unsupervised 2) Squat, 4$\:\times\:$2 min, recovery 2 min, 95% HR_peak_, 3 session per week, 4 week	SBP, DBP, MAP
Wiles 2024	UK	41	56.6	27.3	59	1)Established Hypertension 2) No-medicated	1) Supervised/ Unsupervised 2) Squat, 4$\:\times\:$2 min, recovery 2 min, 95% HR_peak_, 5 session per week, 6 months	SBP, DBP
Xu 2022	Asian	144	73.7	n/a	49	1)Established Hypertension 2) medicated	1) Supervised 2) Squat, 4$\:\times\:$1 min, recovery 0 min, 30% of MVC, 3 session per week, 2 months	SBP, DBP

NOTE: MVC = maximum voluntary contraction; SBP = systolic blood pressure; DBP = diastolic blood pressure; MAP = mean arterial pressure; n/a = not applicable

### Efficacy of IRT on blood pressure

**Fig. 2:**
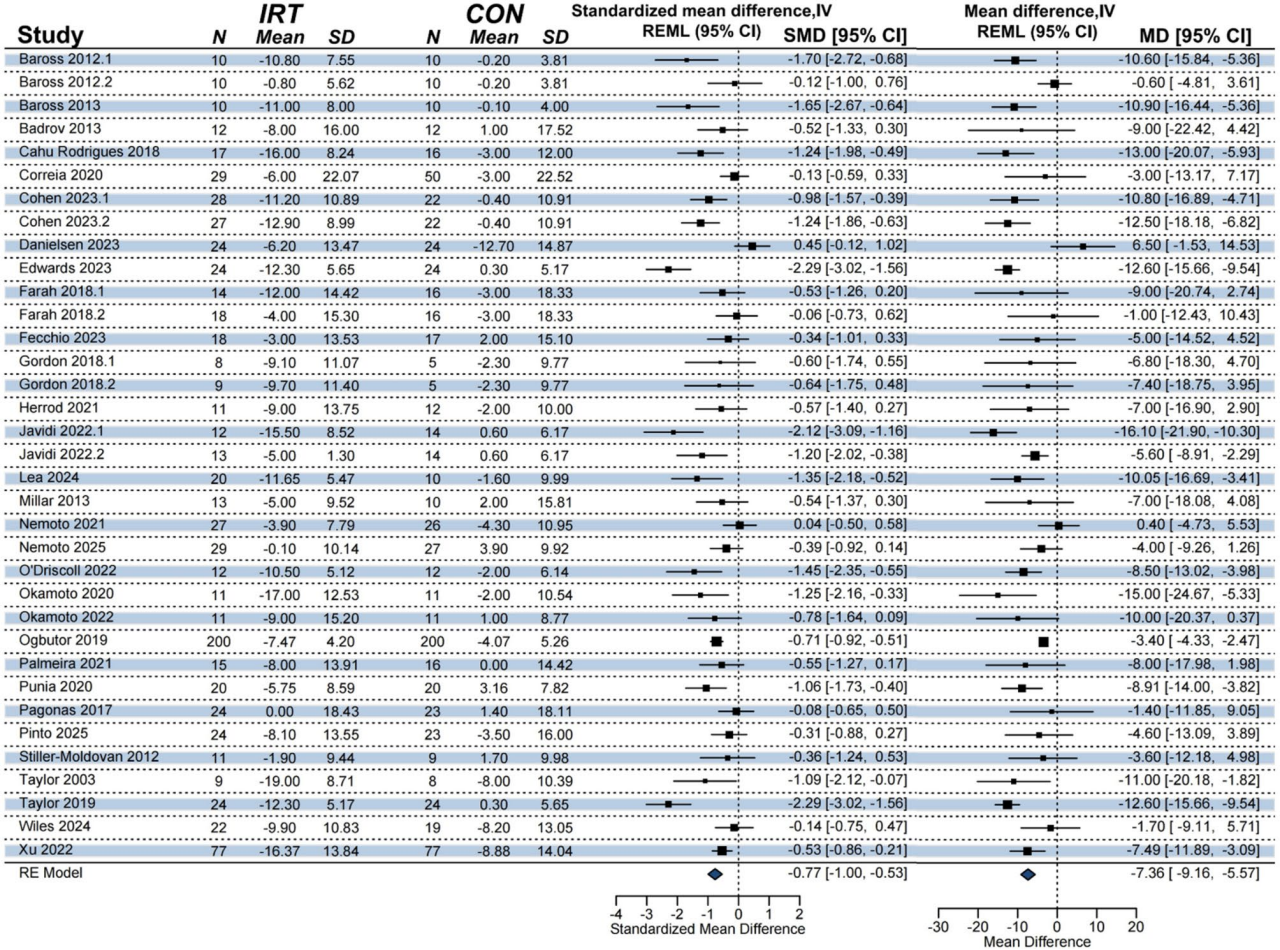
Forest plot of the meta-analysis on the effect of IRT on SBP. NOTE: REML = restricted maximum likelihood estimation; RE model = random-effects model; N = sample size

A meta-analysis including 30 studies found a significant improvement effect of IRT on SBP compared to non- training control (*k* = 35, *n* = 1668, MD = -7.31 mmHg; 95% CI [-9.19, -5.43]; *p* < 0.0001; Fig. 2), with considerable heterogeneity (*I*^2^ = 75.75%, PI [-15.52, 0.89]), this represents a moderate effect size (Hedges’ g = -0.76).

**Fig. 3:**
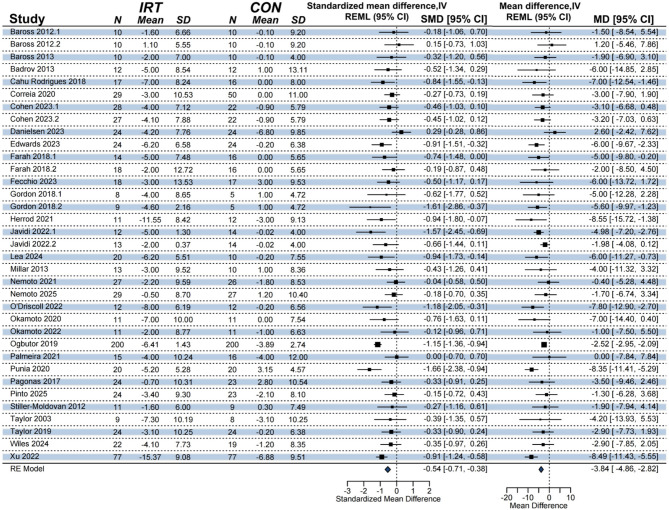
Forest plot of the meta-analysis on the effect of IRT on DBP. NOTE: REML = restricted maximum likelihood estimation; RE model = random-effects model; N = sample size

A meta-analysis including 30 studies found a significant improvement effect of IRT on DBP compared to non- training control (*k* = 35, *n* = 1668, MD = -3.90 mmHg; 95% CI [-4.97, -2.83]; *p* < 0.0001; Fig. 3), with moderate heterogeneity (I^2^ = 48%, PI [-7.50, -0.29]), this represents a moderate effect size (Hedges’ g = -0.54).

**Fig. 4:**
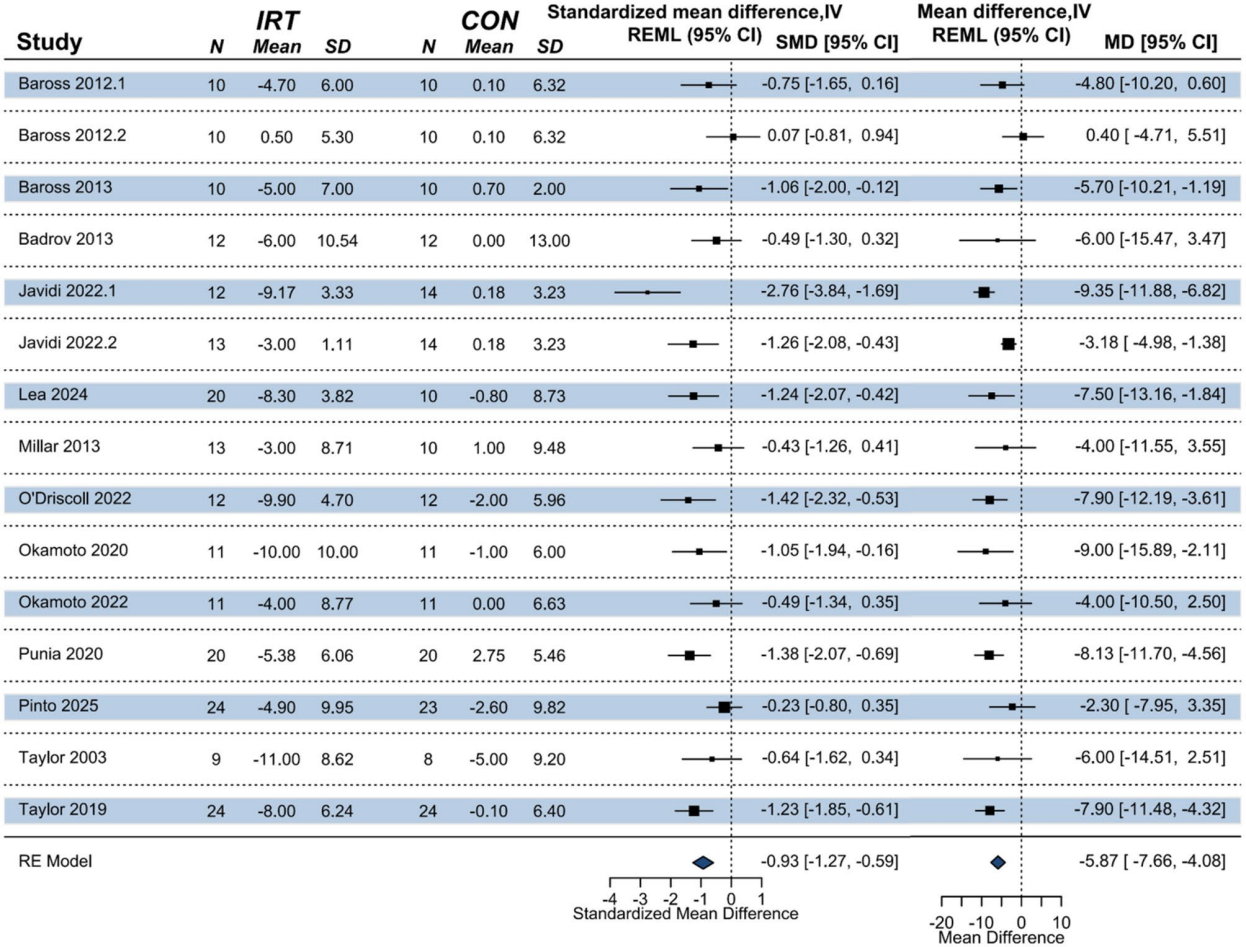
Forest plot of the meta-analysis on the effect of IRT on MAP. NOTE: REML = restricted maximum likelihood estimation; RE model = random-effects model; N = sample size

A meta-analysis including 12 studies found a significant improvement effect of IRT on MAP compared to non- training control (*k* = 15, *n* = 420, MD = -5.94 mmHg; 95% CI [-7.92, -3.96]; *p* < 0.001; Fig. 4), with substantial heterogeneity (I^2^ = 53%, PI [-11.29, -0.59]), this represents a large effect size (Hedges’ g = -0.92). 

**Fig. 5:**
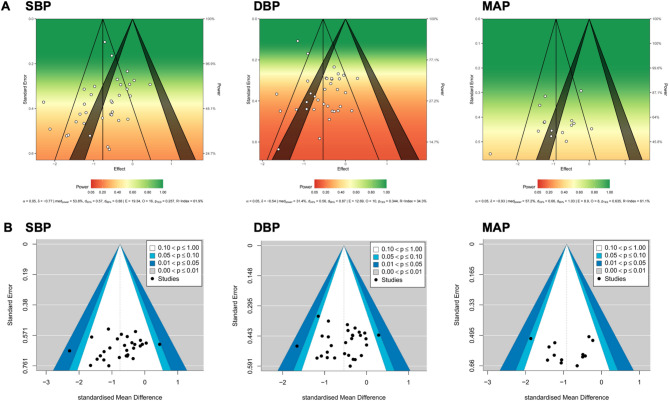
Statistical power and funnel plots

The statistical power of the above main effect estimates is presented in Fig. 5A. Risk of publication bias was assessed using funnel plots (Fig. 5B), and Egger’s test indicated no evidence of publication bias across all outcomes.

### Moderators of IRT on blood pressure

The following are the results (Table 2) of the subgroup analysis of the effect of IRT on SBP compared to no training. We found that region, health conditions, status of medication, training mode, interval duration, and frequency were significant moderators of the effect of IRT on SBP.

**Table 2:** Subgroup analyses based on meta-analyses results of SBP

Subgroup	Sample Size	MD	95% CI	I^2^	*p*_h_	*p*_b_
**Region**						
American	421	-7.34	[-10.42 to -4.26]	38	< 0.01	**< 0.01**
European	447	-7.74	[-10.61 to -4.87]	69	< 0.01	
Asian	400	-7.68	[-11.18 to -4.18]	69	< 0.01	
Africa	400	-3.40	[-4.33 to -2.47]	n/a	n/a	
**BMI**						
Overweight	1194	-6.51	[-8.69 to -4.32]	76	< 0.01	0.08
Obesity	148	-7.78	[-12.15 to -3.41]	9	< 0.01	
Unreported	282	-10.23	[-12.58 to -7.87]	10	< 0.01	
Normal	44	-12.67	[-19.75 to -5.60]	0	< 0.01	
**Health Condition**						
Established Hypertension	894	-6.97	[-9.24 to -4.71]	53	< 0.01	0.63
Pre-hypertension	774	-7.92	[-11.03 to -4.81]	86	< 0.01	
**Status of Medication**						
Yes	1221	-4.98	[-6.79 to -3.17]	31	< 0.01	**< 0.01**
No	447	-9.67	[-12.12 to -7.22]	70	< 0.01	
**Intervention**						
Supervised	1062	-7.85	[-10.08 to -5.62]	67	< 0.01	0.50
Unsupervised	606	-6.55	[-9.56 to -3.54]	72	< 0.01	
**Training mode**						
Hand	1368	-5.72	[-7.65 to -3.80]	56	< 0.01	**< 0.01**
Squat	300	-9.84	[-11.32 to -8.37]	74	< 0.01	
**Unilateral and bilateral**						
Unilateral	708	-7.42	[-10.59 to -4.25]	73	< 0.01	0.98
Bilateral	960	-7.36	[-9.47 to -5.25]	62	< 0.01	
**Interval Duration**						
1 min recovery	577	-5.88	[-8.16 to -3.60]	12	< 0.01	**< 0.01**
4 min recovery	164	-6.57	[-12.69 to -0.46]	81	0.04	
2 min recovery	373	-9.19	[-11.86 to -6.51]	68	< 0.01	
0 min recovery	154	-7.49	[-11.89 to -3.09]	n/a	n/a	
5 min recovery	400	-3.40	[-4.33 to -2.47]	n/a	n/a	
**Frequency**						
3 session per week	1177	-7.50	[-9.34 to -5.65]	62	< 0.01	**< 0.01**
5 session per week	91	-8.98	[-16.79 to -1.18]	44	0.02	
7 session per week	400	-3.40	[-4.33 to -2.47]	n/a	n/a	
**Randomisation**						
Yes	1279	-6.62	[-8.80 to -4.44]	76	< 0.01	0.24
Unclearly	164	-8.88	[-11.67 to -6.09]	0	< 0.01	
NO	225	-10.00	[-14.08 to -5.91]	49	< 0.01	

The following are the results (Table 3) of the subgroup analysis of the effect of IRT on DBP compared to no training. We found that BMI, interval duration, and frequency were significant moderators of the effect of IRT on DBP.

**Table 3:** Subgroup analyses based on meta-analyses results of DBP

Subgroup	Sample Size	MD	95% CI	I^2^	*p*_h_	*p*_b_
**Region**						
**American**	**421**	-3.45	[-4.93 to -1.98]	0	< 0.01	0.10
**European**	**447**	-3.88	[-5.38 to -2.38]	0	< 0.01	
**Asian**	**400**	-4.49	[-6.85 to -2.13]	71	< 0.01	
**Africa**	**400**	-2.52	[-2.95 to -2.09]	n/a	n/a	
**BMI**						
Overweight	1194	-2.63	[-3.02 to -2.24]	0	< 0.01	**< 0.01**
Obesity	148	-3.80	[-6.44 to -1.16]	0	< 0.01	
Unreported	282	-7.74	[-9.49 to -5.99]	0	< 0.01	
Normal	44	-3.73	[-9.58 to -2.13]	30	0.21	
**Health Condition**						
Hypertensive	894	-3.96	[-5.11 to -2.81]	26	< 0.01	0.76
Pre-hypertension	774	-3.65	[-5.35 to -1.95]	53	< 0.01	
**Status of Medication**						
Yes	1221	-3.99	[-5.45 to -2.53]	55	< 0.01	0.68
No	447	-3.60	[-4.66 to -2.54]	2	< 0.01	
**Intervention**						
Supervised	1062	-3.76	[-4.87 to -2.65]	36	< 0.01	0.98
Unsupervised	606	-3.78	[-5.47 to -2.09]	44	< 0.01	
**Training mode**						
Hand	1368	-3.85	[-4.98 to -2.73]	50	< 0.01	0.95
Squat	300	-3.92	[-5.53 to -2.30]	0	< 0.01	
**Unilateral and bilateral**						
Unilateral	708	-3.84	[-5.37 to -2.31]	62	< 0.01	0.86
Bilateral	960	-4.01	[-5.16 to -2.86]	11	< 0.01	
**Interval Duration**						
1 min recovery	577	-3.41	[-4.84 to -1.98]	0	< 0.01	**< 0.01**
4 min recovery	164	-3.62	[-6.78 to -0.46]	79	0.02	
2 min recovery	373	-3.97	[-5.41 to -2.53]	0	< 0.01	
0 min recovery	154	-8.49	[-11.43 to -5.55]	n/a	n/a	
5 min recovery	400	-2.52	[-2.95 to -2.09]	n/a	n/a	
**Frequency**						
3 session per week	1177	-3.99	[-5.04 to -2.94]	34	< 0.01	0.04
5 session per week	91	-3.57	[-7.34 to 0.21]	0	0.06	
7 session per week	400	-2.52	[-2.95 to -2.09]	n/a	n/a	
**Randomization**						
Yes	1279	-3.23	[-4.22 to -2.25]	36	< 0.01	0.09
Unclearly	164	-5.61	[-7.77 to -3.45]	0	< 0.01	
NO	225	-5.74	[-9.77 to -1.71]	54	< 0.01	

No significant subgroup-adjusted analysis results were found for MAP (Table 4).

**Table 4:** Subgroup analyses based on meta-analyses results of MAP

Subgroup	Sample Size	MD	95% CI	I^2^	*p*_h_	*p*_b_
**Region**						
American	64	-5.17	[-10.02 to -0.33]	0	0.04	0.81
European	209	-5.39	[-7.70 to -3.08]	39	< 0.01	
Asian	137	-6.65	[-10.00 to -3.31]	78	< 0.01	
**BMI**						
Overweight	309	-5.52	[-7.48 to -3.57]	60	< 0.01	0.50
Unreported	57	-7.81	[-11.10 to -4.52]	0	< 0.01	
Normal	44	-6.36	[-11.25 to -1.47]	7	0.01	
**Health Condition**						
Hypertensive	163	-6.52	[-9.44 to -3.60]	66	< 0.01	0.55
Pre-hypertension	247	-5.42	[-7.53 to -3.30]	35	< 0.01	
**Status of Medication**						
Yes	151	-6.10	[-8.67 to -3.54]	0	< 0.01	0.92
No	259	-5.93	[-8.07 to -3.79]	64	< 0.01	
**Intervention**						
Supervised	177	-4.90	[-7.55 to -2.25]	65	< 0.01	0.17
Unsupervised	233	-7.15	[-8.92 to -5.38]	0	< 0.01	
**Training mode**						
Hand	248	-5.91	[-8.35 to -3.48]	60	< 0.01	0.95
Squat	162	-5.79	[-8.25 to -3.34]	40	< 0.01	
**Unilateral and bilateral**						
Unilateral	160	-6.38	[-9.42 to -3.34]	73	< 0.01	0.65
Bilateral	250	-5.53	[-7.47 to -3.59]	19	< 0.01	
**Interval Duration**						
1 min recovery	132	-5.01	[-8.16 to -1.86]	0	< 0.01	0.86
4 min recovery	116	-6.36	[-10.15 to -2.57]	83	< 0.01	
2 min recovery	162	-5.79	[-8.25 to -3.34]	40	< 0.01	
**Frequency**						
3 session per week	366	-5.80	[-7.62 to -3.98]	56	< 0.01	0.83
5 session per week	44	-6.36	[-11.25 to -1.47]	7	0.01	
**Randomization**						
Yes	222	-4.96	[-7.44 to -2.48]	70	< 0.01	0.32
Unclearly	117	-7.62	[-10.38 to -4.86]	0	< 0.01	
No	71	-7.18	[-10.42 to -3.95]	0	< 0.01	

**Fig. 6:**
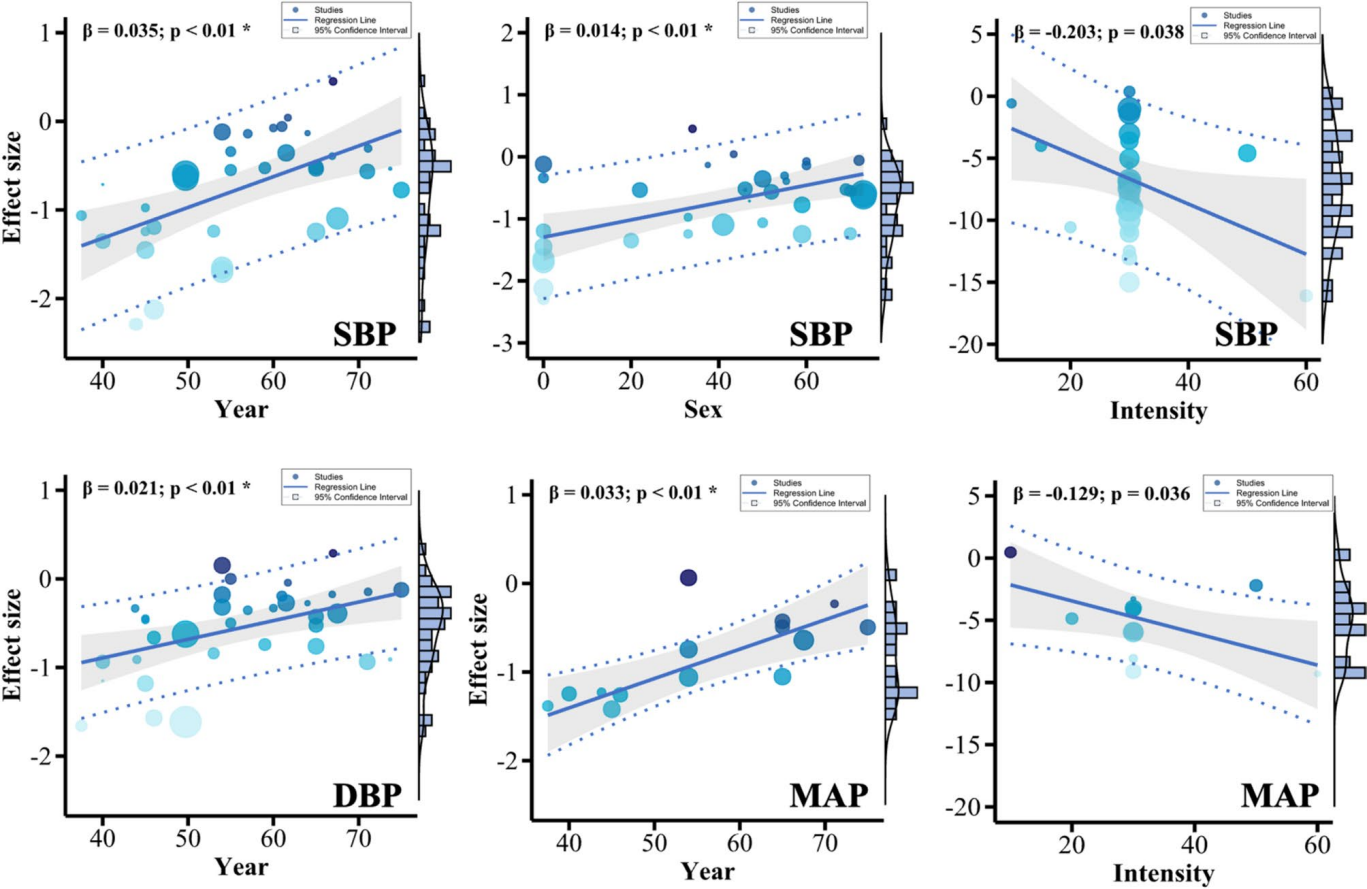
Regression analysis bubble chart. NOTE: "Year" represents the mean age of participants, "Sex" denotes the proportion of women in each study (%), and "Intensity" refers to the %MVC of IRT. The regression analysis for SBP included 29 effects, excluding 6 effects that were quantified using % HRpeak. Similarly, the MAP included 11 effects, excluding 4 effects measured by %HRpeak

Meta-regression analysis (Fig. 6) revealed that the reduction in SBP following IRT was significantly associated with participants’ age (β = 0.04) and the proportion of female participants (β = 0.02). Additionally, higher IRT intensity (%MVC) was significantly associated with greater SBP reductions (β = − 0.20). Specifically, within the range of 10–60% MVC, each 10% increase in intensity was associated with an approximate 2 mmHg reduction in SBP. For DBP, only age emerged as a significant moderator (β = 0.02). Likewise, the reduction in MAP was significantly associated with age (β = 0.03) and IRT intensity (β = − 0.13), with every 10% increase in %MVC being associated with a 1.3 mmHg decrease in MAP. No other training-related variables were found to be significantly associated with changes in SBP, DBP, or MAP (all *p* > 0.05, Supplementary Material 2).

### Risk of bias

**Fig. 7:**
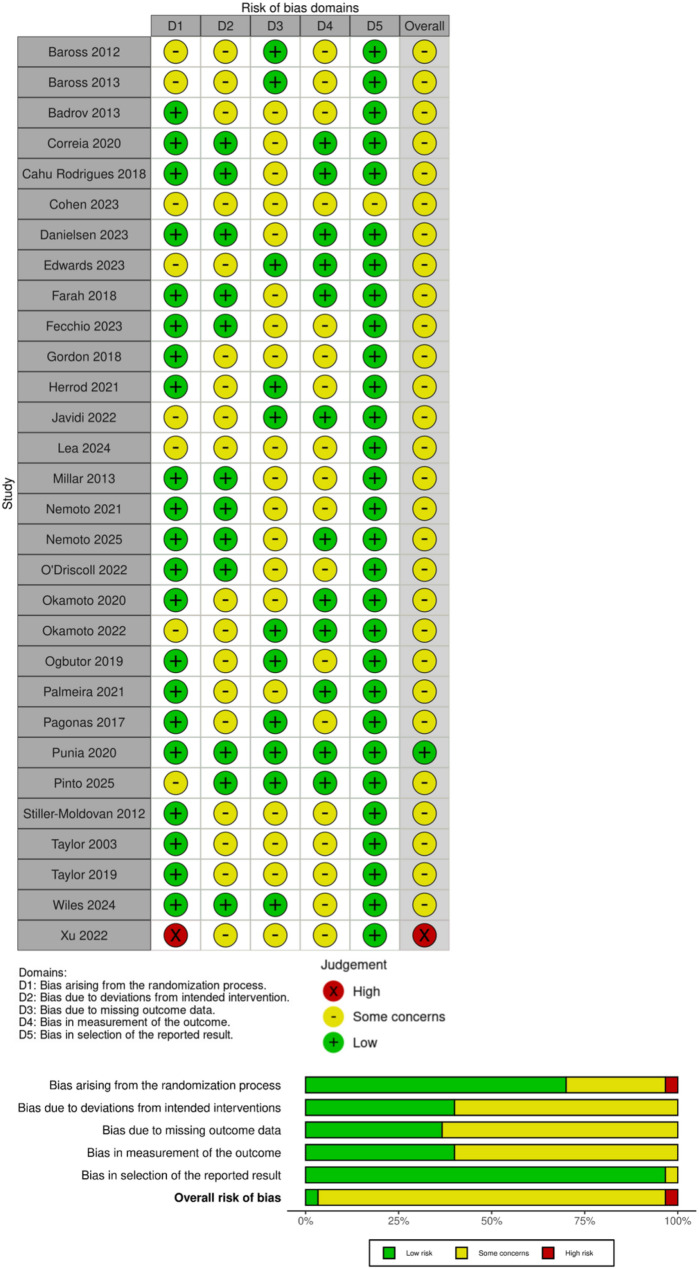
Risk of bias plot

The risk of bias for the methodological design is shown in Fig. 7. The risk of bias for randomization was considered low, but some studies did not implement allocation concealment. As it was not possible to blind participants to exercise therapy, the risk of bias against a possible placebo effect is unlikely to be present.

### Sensitivity analysis

Sensitivity analyses showed that the pooled effect was robust, and the exclusion of any of the studies did not alter the statistical significance of the pooled effect or produce a substantial change in the pooled results (Supplementary Material 3).

### Certainty of evidence

**Fig. 8:**
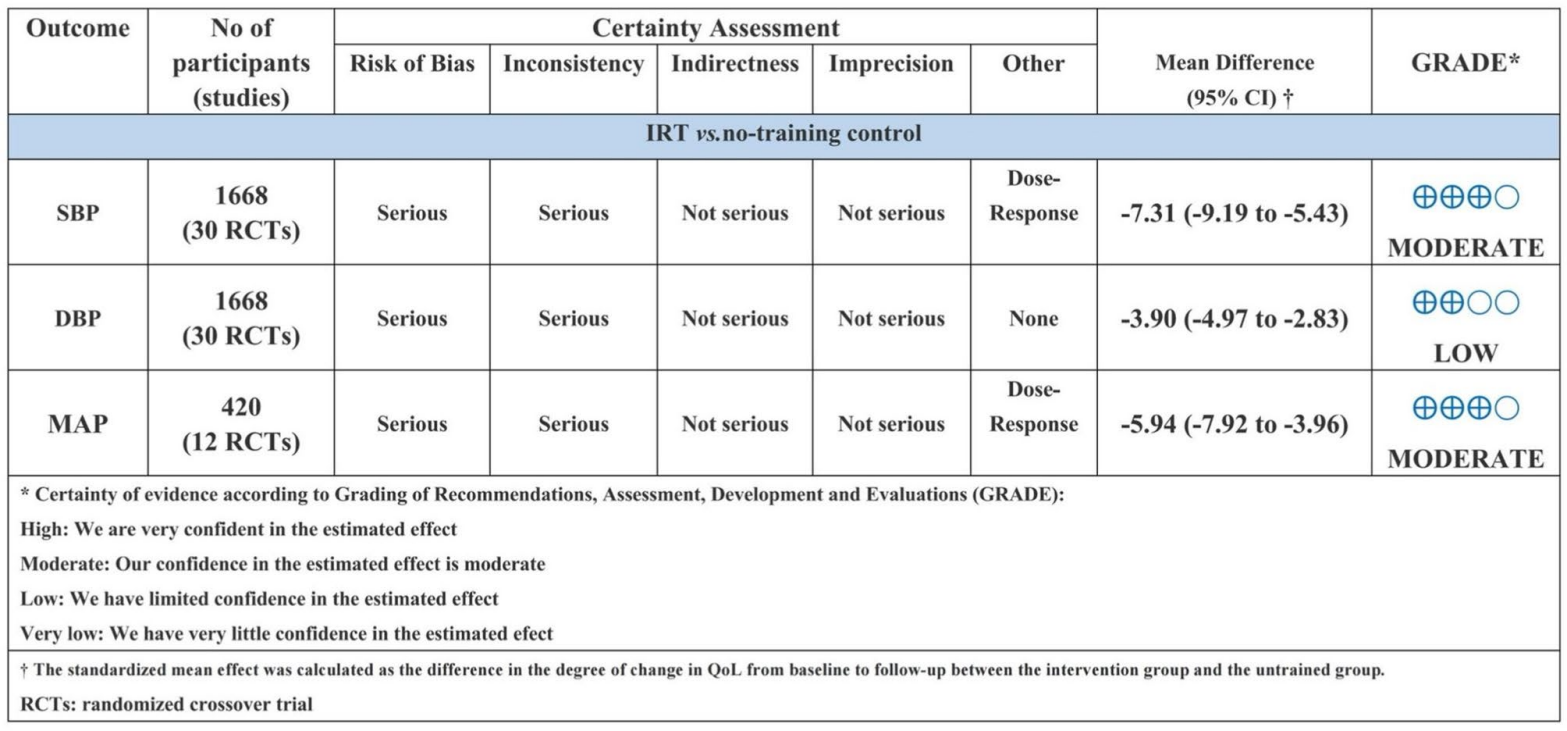
Level of evidence chart

The summary of GRADE results (Fig. 8) provides an evaluation of the certainty of evidence assessment based on the various outcomes analyzed and subsequent performance.

## Discussion

This review is the first meta-analysis to comprehensively evaluate both the effects and moderators of IRT on blood pressure in individuals with pre-hypertension to established hypertension, based on controlled trials. Our main findings showed: (1) Average 11 min (4 × 2 min, recovery 1 min) with 3 sessions per week over 12 weeks of IRT was efficacious in the reduction of SBP (-7.31 mmHg), DBP (-3.90 mmHg), and MAP (-5.94 mmHg) for pre- to established hypertension. (2) Participant characteristics such as age, sex, medication status, and baseline hypertension stage may influence the lowering of blood pressure of IRT. (3) For the first time, we identified that key exercise prescription variables, including training mode, intensity, interval recovery duration, and frequency, might moderate the lowering blood pressure effects of IRT. This exploratory finding highlights the importance of individualized IRT prescriptions in clinical practice and underscores the need for further research to refine and optimize IRT protocols. Collectively, these results provide a strong evidence base to inform future clinical guidelines on hypertension management and prevention using IRT.

### Effects of IRT on blood pressure

For patients with hypertension, recent international guidelines and expert statements based on comprehensive reviews have recommended exercise as a first-line non-pharmacological treatment. Although currently available guidelines and expert consensus recognize the benefits of IRT in preventing hypertension in healthy adults with normal blood pressure [66]. However, there is still a problem of conflicting conclusions from single controlled trials to systematic reviews in adults with pre- to established hypertension. The results of our meta-analysis on IRT demonstrated significant SBP, DBP, and MAP reductions of -7.31, -3.90, and − 5.94 mmHg, respectively. These reductions are clinically substantial and similar to those commonly observed following standard anti-hypertensive pharmacotherapy [67]. In adults with high blood pressure, blood pressure treatment was associated with a 12%-22% reduction in cardiovascular disease risk and a 7%-13% reduction in all-cause mortality [68].

Several previous reviews have established the efficacy of IRT in lowering blood pressure. For instance, Cornelissen and Smart (2013) found that IRT reduced SBP by 10.8 mmHg, and Carlson et al. [27] found that IRT reduced SBP by 7 mmHg. Smart et al. [69] subsequently updated these and showed 7/5 mmHg reductions in SBP/DBP, respectively. However, these previous works were based on highly heterogeneous participants that included normal blood pressure, pre-hypertensive, and hypertensive patients. In 2021, Almeida et al. evaluated the effects of IRT in hypertensive subjects and suggested reduced SBP (-8.11 mmHg) but not DBP (-2.75). However, Smart, (2022) [69] noted some methodological problems with the work of Almeida et al., such as the inclusion of non-randomized controlled trials (which did not meet the review’s inclusion criteria) and the erroneous exclusion of some potential work, which may have been one of the main reasons why no reduction in DBP was observed. Subsequent work has attempted to update the IRT studies in hypertensive populations. Baffour-Awuah et al. [16] included twelve trials that showed reductions in SBP (-7.47 mmHg), DBP (-3.17 mmHg), and MAP (-7.19 mmHg). In contrast, our study incorporated 30 studies with a broader range of participant types and geographical regions. Additionally, we employed rigorous subgroup, regression, and sensitivity analyses to thoroughly examine the sources of heterogeneity and attempt to identify the optimal IRT protocol.

The efficacy of IRT in lowering blood pressure is comparable to other exercise interventions. Current guidelines recommend traditional aerobic continuous exercise, which has been shown to reduce resting blood pressure by 8.9 mmHg in hypertensive individuals [8]. Recent studies indicate that even low-volume HIIT can reduce resting SBP with a 10 mmHg decrease observed, including in patients with metabolic syndrome and hypertension [70]. A recent meta-analysis focusing specifically on patients with metabolic syndrome revealed that HIIT led to reductions in both SBP (-6.05 mmHg) and DBP (-3.68 mmHg) [71]. Some indirect evidence suggests that current IRT is superior to other types of exercise in lowering blood pressure. The largest network analysis to date on blood pressure reduction has shown that isotonic training is the most effective type of exercise, outperforming combined training, dynamic resistance training, aerobic exercise, and HIIT [11]. However, it is important to note that the results from this network meta-analysis provide only indirect evidence, and it covers all populations, so it is not known whether this conclusion also applies to patients with hypertension. In the only meta-analysis directly comparing various exercise types, the researchers found that IRT resulted in significantly greater reductions in resting blood pressure compared to HIIT, showing advantages for SBP, DBP, and MAP of 8.50 mmHg vs. 2.86 mmHg, 4.07 mmHg vs. 2.48 mmHg, and 6.46 mmHg vs. 3.15 mmHg, respectively [12]. Considering the time-saving nature, portability, and clinical efficacy of IRT in lowering blood pressure in hypertensive patients, the potential advantages of IRT over other types of exercise are evident. Lastly, advancements in artificial intelligence have enhanced its application in cardiovascular health, particularly in hypertension management [72]. Combining IRT with artificial intelligence allows for personalized exercise prescriptions, improving blood pressure control, and optimizing cardiovascular outcomes.

IRT has demonstrated significant potential in reducing blood pressure, though the precise physiological and molecular mechanisms remain only partially understood. Evidence suggests that the blood pressure-lowering effect of IRT is primarily mediated through a reduction in systemic vascular resistance, likely linked to diminished sympathetic vascular regulation [27]. Studies have observed minimal changes in cardiac output following IRT, indicating that its primary impact may be on vascular function rather than cardiac output [12]. One hypothesis proposes that IRT enhances endothelial-dependent vasodilation, particularly through nitric oxide (NO)-mediated mechanisms. This effect is especially evident in patients with hypertensive responses to reactive hyperemia, often due to pharmacological treatment [73]. The improvement in endothelial function may contribute to reduced peripheral resistance. Furthermore, research indicates that IRT enhances the function of resistance vessels by increasing arterial diameter, boosting blood flow velocity, and elevating blood volume in trained limbs, collectively suggesting improved vascular conductance [74]. While these functional adaptations, along with improved autonomic vasomotor control, are considered key mechanisms, structural vascular remodeling remains speculative and requires validation through longitudinal studies. Additional physiological pathways may also be involved, including the renin-angiotensin-aldosterone system (RAAS), prostaglandins, and brain natriuretic peptide, although these have not been thoroughly examined [14]. Another contributing factor may be IRT’s association with increased antioxidant levels, which could reduce tissue oxidative stress, enhance endothelial function, improve baroreflex sensitivity, and promote long-term autonomic balance. However, most studies to date have focused predominantly on primary blood pressure outcomes rather than exploring these underlying molecular mechanisms in detail. Further comprehensive clinical trials are necessary to investigate how IRT influences neuroregulatory, inflammatory, and vascular pathways to achieve its blood pressure-lowering effects [16].

### Moderator of IRT on blood pressure

This study elucidated multiple factors that may modulate the effect of IRT on blood pressure, as discerned through subgroup and regression analyses. These factors encompass age, sex, BMI, health conditions, medication status, the direct supervision of the intervention, training intensity, training mode, interval recovery duration, and training frequency. The subsequent sections will delve into the implications of these findings.

#### Moderator effects of participant characteristics

Our meta-regression analyses revealed that participant age was negatively associated with the magnitude of blood pressure reductions following IRT, indicating that younger individuals experienced more pronounced decreases in SBP, DBP, and MAP. This inverse association suggests that age-related physiological changes, such as increased arterial stiffness, impaired endothelial function, and reduced autonomic responsiveness, may attenuate the cardiovascular adaptations elicited by IRT in older populations. These age-related declines in vascular elasticity and neuromuscular plasticity likely limit the hemodynamic benefits of isometric stimuli, thereby reducing the overall efficacy of IRT in older adults [75]. This finding may be attributed to physiological sex differences in neural and cardiovascular responses to isometric exercise. Previous research has shown that women tend to exhibit lower levels of sympathetic nervous system activation during isometric exercise compared to men, resulting in a reduced pressor response [76]. Furthermore, the hemodynamic mechanisms underlying blood pressure elevation during isometric activity differ by sex: women primarily rely on increased cardiac output, while men exhibit increases in both cardiac output and systemic vascular resistance [77]. Although preliminary, this finding highlights the importance of considering age and sex-specific responses when designing and prescribing IRT interventions and calls for further research to elucidate the underlying mechanisms.

The present study demonstrates that the utilization of IRT is notably more effective in reducing SBP in individuals with pre-hypertension compared to those with established hypertension. This finding diverges from previous research, which did not establish a significant moderating effect of baseline blood pressure status on the extent of SBP reduction, due to limited evidence [69]. Interestingly, contrasting findings showed that intermittent high-intensity exercise resulted in greater SBP and DBP reductions in hypertensive participants than in those with pre-hypertension, suggesting that IRT, rather than intermittent high-intensity exercise, may serve as an effective preventive measure for blood pressure management in prehypertensive patients. Furthermore, our study found that individuals not on antihypertensive medication experienced a greater reduction in SBP (-11.26 mmHg) than those on medication (-5.71 mmHg), aligning with findings from Baffour-Awuah et al. [16]. This suggests that the blood pressure-lowering mechanisms of IRT, such as improved endothelial function and reduced sympathetic activity, may overlap with those of certain antihypertensive medications, thereby diminishing the potential for further reductions in individuals already under pharmacological treatment. These interpretations, while provisional, highlight the need for further research to elucidate the underlying physiological mechanisms. Additionally, we found that in terms of study design, studies using strict, explicitly randomized subgroups did not reduce SBP as much as controlled trials that did not use randomized subgroups, which further confirms the potential risk of bias regarding the results related to BP reduction by IRT.

#### Moderator effects of IRT training variables

In the current study concerning intervention protocols, we observed that supervised IRT resulted in a more pronounced reduction of SBP compared to unsupervised IRT, with decreases of 8.07 mmHg and 6.39 mmHg, respectively. This finding is particularly noteworthy as it contradicts the outcomes of several recent unsupervised IRT studies, which did not substantiate its efficacy in lowering blood pressure [22]. Possible explanations include: participants in the IRT group potentially experiencing transient increases in blood pressure due to anticipatory excitement, akin to white-coat hypertension; lower completion and adherence rates in unsupervised settings, as supported by existing literature; and a high likelihood of publication bias, where studies showing no effect of intervention are less frequently published. Our current analysis confirms this bias, echoing the conclusions of prior systematic reviews [69]. Future, unsupervised IRT interventions are urgently needed to verify their ecological validity and long-term feasibility in daily life.

Exercise intensity is a critical determinant of cardiovascular adaptations induced by training. Identifying the minimum effective or optimal intensity threshold for reducing blood pressure is essential to guide the application of IRT for cardiovascular health. Our meta-regression analysis is the first to demonstrate a significant association between IRT intensity (%MVC) and reductions in both SBP and MAP. Specifically, for every 10% increase in MVC within the range of 10–60%, there was an associated reduction of approximately 2 mmHg in SBP and 1.3 mmHg in MAP. These findings are consistent with previous studies indicating that low-intensity interventions (e.g., 10% MVC [49] or 5% MVC [78]) may not be sufficient to elicit meaningful reductions in blood pressure. In contrast, a study by Javidi et al. [18] directly compared protocols at 60% MVC (8 × 30s contractions) and 30% MVC (4 × 2 min) and found that the higher-intensity protocol elicited significantly greater reductions in diastolic blood pressure. These results support the hypothesis that higher training intensities might enhance the antihypertensive effects of IRT. However, it is important to note that our regression findings are based on observational data and do not establish causality. Therefore, further large-scale, well-controlled trials are needed to confirm the independent role of intensity in mediating the antihypertensive effects of IRT.

IRT is most commonly implemented using either upper-limb handgrip or lower-limb static squat exercises. Our subgroup analysis tentatively suggests that static squatting may be associated with greater reductions in SBP compared to handgrip, with observed decreases of 9.84 mmHg for squats versus 5.72 mmHg for handgrip training. This addresses the gap highlighted by Baffour-Awuah et al. [16], who reported inadequate evidence to discern the impact of training modes. The greater magnitude of effect observed with the wall squat might be attributable to differences in the amount of recruited muscle mass and the resulting surface area of compressed vasculature compared to handgrip protocols [79]. Additionally, wall squats involve activation of postural and stabilizing muscles to maintain position, which may differentiate them from leg extension IRT and potentially contribute to enhanced cardiovascular responses [80]. However, these results should be interpreted with caution. The majority of studies involving static squats were derived from a single research group, and the number of included trials was limited. Moreover, most of these interventions were conducted in unsupervised settings. Therefore, current evidence does not permit firm conclusions regarding the superiority of one exercise modality over another. These findings provide only an initial indication that exercise mode might influence the BP-lowering effects of IRT. Future large-scale, well-controlled studies directly comparing handgrip and squat-based IRT are needed to validate this prospective observation. Furthermore, incorporating more diverse exercise formats, such as Tai Chi [81], in combination with IRT may offer benefits in adherence and variety.

In our analysis, a 2-minute recovery interval demonstrated the most significant improvement in SBP; however, no statistical differences were found among the subgroups. On the other hand, DBP showed the most substantial improvement with a 0-minute recovery interval, although this protocol was examined in only one study, which originated in China [46], the recovery interval may be an influential factor in the improvement of blood pressure by IRT. An interesting hypothesis to explore is whether similar health benefits can be achieved when the interval between sessions exceeds one hour (a strategy called exercise snacks) [82], specifically when IRT is incorporated throughout the day. This approach may help address the challenge faced by some patients who struggle to complete multiple sessions in one sitting or remain seated for extended periods. The current lack of variability in training protocols limits the ability to make specific recommendations regarding training duration, intensity, and recovery intervals. Additionally, our regression analyses aimed at identifying dose-response relationships between blood pressure reduction and various aspects of IRT, such as session duration, intervention period, and overall duration, did not reveal any significant predictive factors. These findings underscore the need for longer-term studies to validate these observations. Future research should focus on developing individualized IRT prescriptions tailored to different populations and contexts. Hypertension guidelines should also incorporate the existing evidence on IRT and its recommendations for exercise prescription.

Lastly, Subgroup analysis by training frequency showed between-group differences in both SBP and DBP. Similar effects were observed in the 3-day/week and 5-day/week groups, while the 7-day/week group showed the smallest reductions. This suggests that lower weekly IRT frequency may still drive comparable BP adaptations. These findings highlight the potential of shorter-duration protocols to achieve similar outcomes, aligning with recent interest in minimal effective training doses [83, 84]. Clarifying the lowest effective frequency of IRT could help free up time for other beneficial activities, such as balance or aerobic training [7]. However, the number of studies per subgroup was limited, and results should be interpreted cautiously. Further research directly comparing different IRT frequencies is needed to confirm these initial observations.

### Practical application and future direction

This meta-analysis presents the most up-to-date and comprehensive evidence regarding the efficacy of IRT in individuals with pre-hypertension and established hypertension. It demonstrates that IRT significantly reduces SBP, DBP, and MAP, underscoring its potential as a viable non-pharmacological lifestyle intervention for blood pressure management. Beyond its antihypertensive effects, emerging evidence suggests that IRT may also improve broader cardiovascular health outcomes [14], including vascular function, which is particularly relevant for individuals with elevated cardiovascular risk. From a practical perspective, IRT is highly accessible, cost-effective, and time-efficient. Common formats, particularly handgrip and wall squat exercises—require minimal equipment and space, making them well-suited for home or community settings. A typical session consists of 4 sets of 2-minute contractions with 2-minute rest intervals, totaling approximately 14 min. This structure is feasible even for those with limited time. Based on current evidence, IRT can be recommended at a frequency of 3–5 days per week. For intensity, beginners may start at 20–30% MVC and gradually progress to higher intensities, such as 50–60% MVC, to maximize benefits. The flexibility in mode, dosage, and setting allows IRT to serve not only as a stand-alone intervention but also as a complement to traditional aerobic or resistance training. In the context of increasing sedentary behavior, IRT could also be used intermittently throughout the day to break up prolonged sitting, potentially mitigating its negative impact on cardiovascular and metabolic health. These attributes support the integration of IRT into clinical practice guidelines and public health strategies aimed at preventing and managing hypertension in a scalable and individualized manner.

Several critical gaps in the evidence base remain. First, although this review included trials from multiple continents, only one originated from Africa, while most were conducted in European populations. This geographic imbalance highlights the need for more trials from Africa and other underrepresented or resource-limited regions to improve global generalizability. Second, the majority of included studies had relatively small sample sizes. Future research should prioritize large-scale, multicenter RCTs to strengthen statistical power and external validity. Third, the safety profile of IRT remains insufficiently addressed. Few studies systematically reported adverse events or quantified safety outcomes in hypertensive populations. Similarly, limited data exist on adherence and completion rates of IRT protocols. Future trials should rigorously report safety indicators and adherence metrics to assess the feasibility and real-world applicability of IRT. Fourth, regarding exercise prescription, few studies specified whether the timing of IRT sessions (e.g., morning vs. evening) was standardized. Given growing evidence that exercise timing may affect cardiovascular and metabolic responses, this lack of control may introduce heterogeneity. Future research should investigate the optimal timing of IRT to enhance its clinical effectiveness. Additionally, individual response variability and the comparative effectiveness of different IRT protocols remain poorly understood. Comparative trials are needed to assess variations in training mode (e.g., handgrip vs. wall squat), intensity, and dose-response relationships (e.g., low- vs. high-volume protocols). Furthermore, the combined effects of IRT with traditional aerobic exercise, emerging modalities, and dietary strategies [85] such as blood flow restriction training [86] and ischemic pre-conditioning, should be explored to determine whether these combinations amplify physiological benefits. Finally, beyond efficacy and safety, future studies should incorporate mixed-method approaches, including semi-structured interviews, to identify facilitators and barriers to IRT implementation in clinical and community settings. Understanding patients’ perceptions, preferences, and adherence challenges is essential to developing more sustainable, individualized, and scalable exercise prescriptions.

### Strengths and limitations of the review

This study has several strengths, including the inclusion of trials involving both pre-hypertensive and hypertensive populations and the use of subgroup and meta-regression analyses to investigate potential sources of heterogeneity. These approaches build upon previous research and offer more nuanced insights into the practical application of IRT. In addition, several preliminary exploratory findings, such as potential moderators of IRT training variables, may inform future research. However, several limitations should be acknowledged. First, the literature search was restricted to English and Chinese publications, which may have excluded relevant studies in other languages. Second, despite subgroup, sensitivity, and regression analyses, substantial heterogeneity remains in intervention protocols, study designs, and outcome measures, thereby complicating interpretation. Inconsistencies in methodological quality, such as randomization procedures, supervision, and measurement accuracy, may also have introduced bias. Particularly, findings from the squat-based IRT subgroup warrant cautious interpretation. This subgroup included a small number of studies, all conducted by a single research group, limited to pre-hypertensive individuals not receiving antihypertensive treatment, and involving unsupervised training. These issues raise concerns regarding selection, publication, and performance bias, which may compromise both internal validity and generalizability. While early results suggest potential benefits of squat-based IRT, these findings remain exploratory and should be interpreted as tentative. Finally, the possibility of publication bias and unreported confounders must be considered when translating these findings into clinical or public health recommendations. Future studies should aim to replicate these results through well-designed, independently conducted trials involving diverse populations and supervised interventions to better determine the efficacy of IRT and reduce bias.

## Conclusion

IRT can significantly reduce resting SBP, DBP, and MAP in people with pre- to established hypertension, which might be incorporated into community health programs as a cost-effective strategy to manage blood pressure and prevent hypertension progression. However, the magnitude of blood pressure reduction may vary across populations. Factors such as age, sex, medication status, and baseline blood pressure level are likely to act as potential moderators of the antihypertensive effects of IRT, highlighting the need for precise control of these variables in both clinical practice and future research. The exercise prescription variables of IRT, such as intensity, modality, and recovery intervals, might influence its blood pressure-lowering efficacy. Based on the current evidence and our findings, we recommend handgrip IRT at 30–60% MVC (gradually increase) or wall squat IRT at approximately 85%-95% HR_peak_, performed 3–5 sessions per week for 14 min per session (4 × 2-minute contractions with 2-minute recovery). These protocols appear effective in optimizing resting BP reduction. Nonetheless, individual customization based on health status, accessibility, and adherence should be considered to ensure safety and maximize effectiveness in real-world settings.

## Supplementary Information

Below is the link to the electronic supplementary material.

Supplementary Material 1

## Data Availability

The datasets used and/or analysed during the current study are available from the corresponding author upon reasonable request.

## Abbreviations

SBP: Systolic Blood Pressure
DBP: Diastolic Blood Pressure
MAP: Mean Arterial Pressure
HIIT: High-Intensity Interval Training
MICT: Moderate-Intensity Continuous Training
IRT: Isometric Resistance Training
MD: Mean Difference
SMD: Standardized Mean Difference
MVC: Maximum Voluntary Contraction
NO: Nitric Oxide
